# Studies on Pathogen Identification, Biological Characteristics and Fungicide Sensitivity of *Impatiens hawkeri* Leaf Spot Disease

**DOI:** 10.3390/jof12030210

**Published:** 2026-03-14

**Authors:** Mengyao Wang, Ziyue Zhang, Yajiao Sun, Huali Li, Jian Liu, Shuwen Liu, Yunqiang Ma, Junjia Lu

**Affiliations:** 1 College of Landscape Architecture and Horticulture Sciences, Southwest Forestry University Sciences, Kunming 650224, China; 17587020327@163.com (M.W.); 18313871472@163.com (Z.Z.); 18087323192@126.com (Y.S.); 15912938064@163.com (H.L.); jian927520@163.com (J.L.); 15887642939@163.com (S.L.); 2 Yunnan Key Laboratory of Forest Disaster Warning and Control, Southwest Forestry University, Kunming 650224, China; 3 Yunnan Key Laboratory of Landscape Plant Resource Cultivation and Application, Southwest Forestry University, Kunming 650224, China; mayunqiang@swfu.edu.cn

**Keywords:** *Impatiens hawkeri*, *Ectophoma multirostrata*, molecular identification, biological characteristics, fungicide sensitivity, infection process

## Abstract

*Impatiens hawkeri* W. Bull (*I. hawkeri*) is popular among consumers due to its diverse flower colors and year-round blooming. However, changes in ecological conditions, cultivation methods, and planting scale have led to increased disease incidence and diversity, particularly the widespread and destructive leaf spot disease. Currently, studies addressing the pathogen species and its biological characteristics remain limited. In this study, a highly pathogenic strain (IH-4) was selected from previously isolated fungi associated with leaf spot in *I. hawkeri*. Its taxonomic status was confirmed using upright fluorescence microscope analysis, internal transcribed spacer (ITS)/large subunit (LSU)/RNA polymerase II second largest subunit (*rpb2*)/β-tubulin (*tub2*) rRNA gene sequencing, and phylogenetic tree construction. Additionally, the biological characteristics of the pathogen and its sensitivity to 8 chemical fungicides were assessed. Strain IH-4 was identified as *Ectophoma multirostrata* (*E. multirostrata*) through combined morphological and molecular approaches. Optimal growth conditions included a temperature of 25 °C, a pH of 7, Potato Dextrose Agar (PDA) medium, fructose as the optimal carbon source, and urea as the optimal nitrogen source, with the fastest growth observed under a semi-light photoperiod (12 h light/12 h dark). Fungicide sensitivity assays indicated that 25% azoxystrobin exhibited the lowest half-maximal effective concentration (EC_50_, 0.0724 μg/mL) and the steepest virulence regression slope (1.7), demonstrating the strongest inhibitory activity and highest sensitivity. Microscopic observations revealed that IH-4 hyphae penetrate *I. hawkeri* leaf tissues via stomata, colonize internally, and consequently cause host damage. This study provides a theoretical foundation for the timely and effective management of leaf spot disease in *I. hawkeri*.

## 1. Introduction

*Impatiens hawkeri* W. Bull, commonly known as New Guinea impatiens [1], is a perennial evergreen herbaceous flowering plant belonging to the genus *Impatiens* (family Balsaminaceae). The family Balsaminaceae comprises two genera, *Impatiens* and *Hydrocera*, with the latter containing only one species. First described by Linnaeus in 1753, *Impatiens* is the largest genus within this family, encompassing over 900 species worldwide [2,3]. These species predominantly occur in tropical and subtropical regions, although a small number are found in temperate zones. Native to New Guinea and the Solomon Islands, *I. hawkeri* is cultivated in parts of Dongguan, China, and has been introduced internationally as a popular greenhouse ornamental. Since its introduction to China as an ornamental plant in the 1990s, *I. hawkeri* has rapidly gained popularity throughout the country. Despite its relatively brief cultivation history abroad, long-term artificial propagation has resulted in several elite varieties distinguished by diverse flower and leaf colors. These improved lines exhibit significant enhancements over the original species, particularly in color diversity, branching capacity, disease resistance, and stress tolerance.

*Impatiens hawkeri* is valued for its distinctive leaf color and shape, glossy foliage, extended flowering period, vivid and varied flower colors, strong adaptability, and ease of shaping. Consequently, it is highly regarded as an ornamental plant suitable for both foliage and flower appreciation. It is extensively used in landscaping, household decoration, and garden beautification, and in recent years has become a favored choice for small potted plants [4]. Globally appreciated by consumers, it holds a significant position in the international horticultural market. Beyond ornamental uses, species within the genus *Impatiens* have notable medicinal applications. Among the more than 20 medicinal materials documented in the *Compendium of Chinese Materia Medica*, approximately one-third are derived from flowers, and *Impatiens* is frequently employed as a medicinal herb. Additionally, *Impatiens* species contain pigment constituents such as malvidin, cyanidin, pelargonidin, and delphinidin [5], classifying them as natural pigment plants. Furthermore, *Impatiens* serves as an important industrial flower with potential applications in the food industry, cosmetics, gold extraction, and wastewater treatment following pigment extraction and purification.

With the expansion of cultivation areas and advances in cultivation techniques for *I. hawkeri*, both disease diversity and severity have increased, resulting in substantial economic losses. In recent years, leaf spot disease has been reported as one of the most widespread and damaging diseases affecting this plant. Characteristically, leaf spot disease manifests as lesions 1–4 mm in diameter, initially circular or subcircular and brown, subsequently turning grayish-white or grayish-brown with distinct brown margins. Severe infections can lead to lesion coalescence, resulting in leaf necrosis [4]. Caused by fungal pathogens, leaf spot primarily infects plants via wounds or natural openings (e.g., stomata), resulting in leaf tip necrosis and scorching along leaf margins. The pathogen overwinters as hyphae on infected leaf debris, subsequently producing abundant conidia that disperse via rainwater, particularly through splash dispersal, to initiate new infections in the following season [6].

Pathogens causing leaf spot diseases are primarily affiliated with the phyla Ascomycota, Basidiomycota, and Proteobacteria, among others. Among these, fungal leaf spot diseases are the most prevalent, with the predominant causal agents belonging to the genera *Alternaria*, *Cercospora*, *Septoria*, *Phyllosticta*, and *Didymella*. Reported pathogens causing leaf spot diseases in plants include *Didymella segeticola* [7], *Alternaria tenuissima* [8], *Corynespora cassiicola* [9], *Nigrospora sphaerica* [10], *Alternaria alternata* [11], and *Cercospora apii* [12].

Previous studies demonstrated that certain species of the genus *Allophoma* (family Didymellaceae) induce leaf spots and necrosis in multiple host plants across Italy, India, Western Europe, and various tropical and subtropical regions globally [13,14], including leaf spot on broccoli [15,16]. Species of the genus *Phoma* infect *Ajuga multiflora*, causing black leg disease; *Musa* spp., causing sheath rot; and *Scutellaria baicalensis*, causing stem blight [15,17,18,19]. *Ascochyta sorghi* is the causal agent of leaf spot on sorghum [20]. *E. multirostrata* has been reported to cause root rot diseases in celosia (*Celosia cristata*), chickpea (*Cicer arietinum*), and mung bean (*Vigna radiata*) [21,22]. Additionally, fungi of the genus *Stagonosporopsis* (Didymellaceae) induce stem blight and black rot in cucurbits, and most species within this genus cause diverse plant diseases worldwide [23,24,25], such as wilt, stunting, leaf spot, and flower spot diseases on Asteraceae plants [26,27,28,29], and leaf spot, stem rot, and leaf blight in various crops [30]. Collectively, fungi from Didymellaceae pose significant threats to plant health and development, leading to substantial losses in quality and yield.

Although well-developed theories and methods exist internationally for characterizing the biological properties of plant pathogens, research concerning the biological characteristics of *E. multirostrata* is currently limited and fragmented. Studies addressing leaf spot disease on *I. hawkeri* remain scarce, and the infection process of the pathogen has not yet been documented at the microscopic level. To address these knowledge gaps, this study systematically investigated the biological characteristics and infection mode of *E. multirostrata*. Mycelial growth was evaluated on Potato Dextrose Agar (PDA) under varied conditions—including carbon sources, nitrogen sources, pH levels, culture media, light regimes, and temperatures—to determine optimal growth parameters and elucidate disease occurrence patterns. In parallel, the inhibitory effects of eight routine fungicides were assessed using the poisoned food technique, and the most effective compound was identified. Moreover, the infection process was examined by inoculating healthy leaves of *I. hawkeri* with mycelial plugs and observing under light microscopy. This approach enabled the visualization of spore germination, fungal penetration, and hyphal expansion within host tissues, offering cytological insights into the pathogen–host interaction. These findings contribute to a better understanding of the infection mechanism and support the development of targeted management strategies for this emerging disease.

## 2. Materials and Methods

### 2.1. Materials

Plant Materials: Leaves of *Impatiens hawkeri* exhibiting typical leaf spot symptoms were collected from the campus of Southwest Forestry University (Kunming, Yunnan Province, China). The fungal strain tested in this study was isolated from these symptomatic leaves.

Test Media: The following culture media were employed in this study: Potato Dextrose Broth (PDB), PDA, Potato Sucrose Agar (PSA), Water Agar (WA), 1/2 Murashige and Skoog (1/2 MS) medium, Oatmeal Agar (OA), Corn Meal Agar (CMA), and Czapek Dox Agar (CDA). All media were sterilized by autoclaving (103.4 kPa) at 121 °C for 20 min prior to use. The detailed components of each culture medium can be found in the Supplementary Materials (Table S1).

### 2.2. Pathogenicity Assay of the Pathogenic Fungus

Strain IH-4 was previously isolated by our research group from infected leaves of *I. hawkeri*. A total of five strains were isolated and purified, among which three were identified as pathogenic through pathogenicity tests. For this study, the IH-4 strain, which exhibited the strongest pathogenicity, was selected for all subsequent experiments and cultured on PDA medium at 28 °C for 5–7 days. The pathogenicity of strain IH-4 to *I. hawkeri* was verified according to Koch’s postulates, using both detached leaves and intact plants [31].

Detached Leaf Inoculation: Healthy *I. hawkeri* leaves were collected, rinsed under running tap water, and air-dried. Surface sterilization was performed by sequential immersion in 75% (*v*/*v*) ethanol for 30 s and 2% (*v*/*v*) sodium hypochlorite (NaClO) for 2 min, followed by three rinses with sterile distilled water [32]. Excess moisture was removed using sterile filter paper. Sterile scalpels were used to create small wounds on each sterilized leaf. A 6 mm diameter mycelial disc, excised from the margin of 7-day-old IH-4 colonies using a sterile cork borer, was placed directly onto each wound, mycelial side down [31]. For controls, sterile PDA discs without fungal mycelium were placed onto wounded leaves. Inoculated leaves were then incubated at 28 °C for 5–7 days in sterile Petri dishes lined with sterile filter paper moistened with sterile distilled water to maintain high humidity. Each treatment was replicated three times. Fungi were subsequently re-isolated from symptomatic lesions, purified through multiple subcultures on PDA medium, and compared morphologically with the original IH-4 strain.

Intact Plant Inoculation: Pathogenicity was determined using one-year-old healthy impatiens plants from *I. hawkeri*. 6 mm diameter mycelial discs from strain IH-4 were directly inoculated onto healthy leaves positioned in the middle canopy of intact *I. hawkeri* plants. This treatment was repeated three times. The inoculated plants were maintained under ambient environmental conditions in Kunming, Yunnan Province, China. During the experimental period, temperatures ranged from 17 to 26 °C (averaging approximately 21 °C), and relative humidity was maintained between 65% and 80%. Natural light was used, with light intensity adjusted to 15,000–25,000 lux using shade nets to accommodate the semi-shaded growth requirements of *I. hawkeri*. Symptom development was monitored daily, and disease progression was regularly documented through photography.

### 2.3. Morphological Identification of the Pathogenic Fungus

The pathogenic strain IH-4 was inoculated onto PDA medium and incubated at 28 °C for 5–7 days in a constant-temperature incubator. Colony morphology, specifically growth rate, color, texture, and margin shape, was observed and recorded daily. Upon conidiation, a small portion of mycelium was excised from colony margins using a sterile dissecting needle, mounted in lactophenol trypan blue stain on a glass slide, and examined under an upright fluorescence microscope (Olympus BX53, Olympus Corporation, Tokyo, Japan). Morphological features of hyphae and conidia (shape, size, color, and septation) were documented, and micrographs were captured for subsequent analysis [31,33].

### 2.4. Molecular Identification of the Pathogenic Fungus

Pathogenic fungi cultured for 7 days exhibiting vigorous growth were selected, and an appropriate amount of mycelium was transferred into sterile 1.5 mL centrifuge tubes. Genomic DNA was extracted using the DNeasy Plant Mini Kit (Qiagen, Hilden, Germany; Cat. No. 69106) following the manufacturer’s instructions. Following extraction, the internal transcribed spacer (ITS), large subunit ribosomal RNA (LSU), RNA polymerase II subunit (*rpb2*), and β-tubulin gene (*tub2*) regions were amplified by polymerase chain reaction (PCR) using an Applied Biosystems 2720 Thermal Cycler (Thermo Fisher Scientific, Waltham, MA, USA). The primers used for amplification are listed in Table 1.

PCR amplification was performed using a Bio-Rad T100 thermal cycler (Bio-Rad, Hercules, CA, USA). Each 25 µL reaction mixture contained the following components: 1 µL forward primer (0.4 µmol/L), 1 µL reverse primer (0.4 µmol/L), 2 µL genomic DNA template (50 ng/µL), 10 µL 1× PCR Master Mix, and 11 µL nuclease-free double-distilled water (ddH_2_O). The optimized thermal cycling conditions were as follows: initial denaturation at 95 °C for 3 min; 36 cycles of denaturation at 95 °C for 40 s, annealing at 55 °C for 40 s, and extension at 72 °C for 45 s; and a final extension at 72 °C for 10 min [34].

PCR products were sequenced by the Kunming Branch of Beijing Tsingke Biotech Co., Ltd. (Kunming, China). The resulting sequences were assembled using DNAMAN software (version 10.0) and subjected to sequence similarity analysis using BLAST in the NCBI database (https://blast.ncbi.nlm.nih.gov/Blast.cgi, accessed on: 13 March 2026) to determine the genus and species of the pathogen. Multiple sequence alignments of the ITS, LSU, rpb2, and tub2 gene sequences were performed separately using the MAFFT program (version 7.x). The L-INS-i strategy was employed with --maxiterate 1000 to ensure high alignment accuracy, as this method is suitable for datasets with fewer than 200 sequences and uses local pairwise alignment with iterative refinement. The resulting alignments were trimmed using Gblocks (version 0.91b) to eliminate poorly aligned and divergent regions, with the parameter setting allowing for gaps in the final alignment blocks. The trimmed alignments for each gene were exported in FASTA format.

Sequence concatenation was conducted using R (version 4.x) with the Biostrings package (version 2.62). Specifically, the *readDNAStringSet* function was used to import the trimmed alignment files for the four genes. The intersect function was employed to ensure that all samples were present and that their identifiers (IDs) were consistent across the four datasets. Subsequently, the four gene fragments for each sample were concatenated end-to-end in the order ITS-LSU-*rpb2*-*tub2* to generate a combined supermatrix, which was then exported in FASTA format.

Based on this concatenated supermatrix, the best-fit nucleotide substitution model was selected using MEGA 12.0. The Akaike Information Criterion (AIC) was applied to evaluate all possible models, and the Tamura-Nei 93 (TN93) + Gamma distribution (+G) model was determined to be the best-fitting model. Accordingly, a phylogenetic tree was reconstructed using the maximum likelihood method implemented in MEGA 12.0 under the TN93 + G model. Nodal support was assessed with 1000 bootstrap replicates.

**Table 1:** PCR primers used in this study.

Gene	Primers	Sequence
ITS [35]	ITS1	5′-TCCGTAGGTGAACCTGCGG-3′
	ITS4	5′-TCCTCCGCTTATTGATATG-3′
LSU [36]	NL1	5′-GCATATCAATAAGCGGAGGAAAAG-3′
	NL4	5′-GGTCCGTGTTTCAAGACGG-3′
*rpb2* [37]	fRPB2-5F2	5′-GGGGWGAYCAGAAGAAGGC-3′
	fRPB2-7cR	5′-CCCATRGCTTGYTTRCCCAT-3′
*tub2* [38]	T1	5′-AACATGCGTGAGATTGTAAGT-3′
	Bt2b	5′-ACCCTCAGTGTAGTGACCCTTGGC-3′

### 2.5. Biological Characteristics of the Pathogenic Fungus

#### 2.5.1. Effect of pH on Mycelial Growth

The pH of PDA medium was adjusted to 5, 6, 7, 8, 9, 10, and 11 [39] using 1 mol/L HCl/NaOH, autoclaved (103.4 kPa, 121 °C, 20 min), cooled to ~60 °C, and poured into sterile 90 mm Petri dishes. A 6 mm diameter mycelial disc from a 5–7-day-old colony cultured at 28 °C was inoculated centrally onto each plate, with three replicates per pH level. Plates were sealed and incubated (28 °C, dark, 5 d), with daily checks for contamination.

#### 2.5.2. Effect of Carbon Sources on Mycelial Growth

Using Czapek Dox agar as the basal medium, equal masses of soluble starch, mannitol, fructose, lactose, sucrose, and maltose were individually used as the sole carbon source to replace the glucose in the medium. The amount of each carbon source added was calculated based on its carbon content to ensure a consistent total carbon concentration across all treatment groups [40]. The sterilization conditions for the medium, as well as the inoculation and culture methods for the pathogenic fungus, were the same as those described in Section 2.5.1. Three replicates were conducted for each carbon source.

#### 2.5.3. Effect of Nitrogen Sources on Mycelial Growth

Using Czapek Dox agar as the basal medium, the original nitrogen source (sodium nitrate) was replaced with equimolar nitrogen amounts of ammonium chloride, yeast extract powder, potassium nitrate, urea, glycine, peptone, or ammonium sulfate, each serving as the sole nitrogen source. The quantity of each nitrogen source added was calculated based on its nitrogen content to ensure a consistent nitrogen element concentration across all treatments [41]. The medium sterilization, fungal inoculation, and culture conditions were the same as those described in Section 2.5.1. Three replicates were performed for each nitrogen source.

#### 2.5.4. Effect of Temperature on Mycelial Growth

The sterilization conditions for the PDA medium, as well as the inoculation and culture methods for the pathogenic fungus, were the same as those described in Section 2.5.1. Three replicates were performed for each temperature treatment (5, 10, 15, 20, 25, 30, and 35 °C) [42].

#### 2.5.5. Effect of Light Conditions on Mycelial Growth

The sterilization conditions for the PDA medium, as well as the inoculation and culture methods for the pathogenic fungus, were the same as those described in Section 2.5.1. Three replicates were performed for each light condition: continuous light, continuous darkness, and a 12 h light/12 h dark photoperiod (2000 lx) [43].

#### 2.5.6. Effect of Culture Media on Mycelial Growth

Seven different media (PDA, WA, PSA, 1/2 MS, OA, CMA, and CDA) were individually sterilized (103.4 kPa, 121 °C, 20 min) [44], cooled, and dispensed into sterile Petri dishes. A 6 mm fungal disc was centrally inoculated onto each plate, with 3 replicates per medium.

### 2.6. Sensitivity of the Pathogenic Fungus to Fungicides

#### 2.6.1. Principles for Fungicide Selection

Fungicides for testing were selected based on two criteria: (1) significant inhibitory or regulatory effects on pathogen growth and development; (2) enhancement of disease resistance in the host plant (*I. hawkeri*).

#### 2.6.2. Types of Fungicides Tested

Detailed information on tested fungicides is presented in Table 2.

#### 2.6.3. Fungicide Concentration Gradients

The concentration gradients of the fungicides are shown in Table 3.

#### 2.6.4. Effect of Fungicides on Mycelial Growth

##### Preparation of Fungicide Stock Solutions and Fungicide-Amended Media

Stock solutions of the 8 fungicides at different concentrations (Table 3) were prepared using sterile distilled water and stored at 4 °C in amber sterile centrifuge tubes (to prevent photodegradation) for no longer than 24 h before use.

PDA medium was autoclaved (103.4 kPa, 121 °C, 20 min), cooled to ~50 °C (to prevent thermal degradation of fungicides), and combined with diluted fungicide stock solutions to achieve the target concentrations (Table 3). For the control, an equivalent volume of sterile distilled water (without fungicide) was added to the PDA medium. To prevent bacterial contamination, 100 μL of streptomycin solution (50 g/L) was added per 100 Ml of medium (treatment and control). Media were poured into sterile Petri dishes under aseptic conditions in a laminar flow hood, and three replicates per fungicide concentration were established. Media were solidified before inoculation.

##### Inoculation and Incubation

A 6 mm mycelial disc from the margin of a 5–7-day-old colony cultured at 28 °C was inoculated centrally onto each fungicide-containing PDA plate, with the mycelial side facing downward. Plates were sealed with breathable Parafilm, labeled (fungicide type, concentration, and date), and incubated (28 °C, dark, 5 d). Daily inspections were conducted, and contaminated plates were discarded immediately.

##### Growth Measurement and Toxicological Analysis

After 5 d of incubation, colony diameters were measured via the cross method (average of two perpendicular diameters, ±0.1 mm). The mycelial growth rate (mm/d) was calculated as follows: Growth rate = (Colony diameter-Disc diameter)/5 [11]. The inhibition rate (%) was calculated as follows: Inhibition rate (%) = [(Control growth rate − Treatment growth rate)/Control growth rate] × 100.

For toxicological analysis, probit values of inhibition rate (Y) were plotted against the logarithm of fungicide concentration (X, μg/mL). SPSS 27.0 was employed for Probit regression analysis to obtain the virulence equation (Y = aX + b), coefficient of determination (R^2^), and half-maximal effective concentration (EC_50_) values (95% confidence intervals [CIs]). Fungicides exhibiting lower EC_50_ values (lower = stronger inhibition) and higher regression slopes (higher = more consistent pathogen sensitivity) were selected as optimal.

### 2.7. Observation of the Infection Process of Pathogenic Fungus IH-4 on I. hawkeri Leaves

#### 2.7.1. Strain Culture and Inoculation

The IH-4 strain was cultured on PDA (28 °C, 7 d) to induce conidiation. Subsequently, the strain was transferred to PDB and incubated on a shaker (25 °C, dark, 7 d, 150 rpm) to increase conidial production. Conidial suspensions were adjusted to 1 × 10^5^ conidia/mL using sterile distilled water, with concentrations verified using a hemocytometer.

Healthy *I. hawkeri* leaves were rinsed, and 10 μL of conidial suspension was pipetted onto both sides of the main leaf vein (2 inoculation points/leaf). Control leaves were treated identically with sterile distilled water. All inoculated plants were maintained in a growth chamber (28 °C, 12 h light/12 h dark photoperiod, 2000 lx, 85% RH) to facilitate infection.

#### 2.7.2. Preparation of Samples for Light Microscopy

Leaf samples were collected at 0, 6, 12, 24, 48, and 72 h post-inoculation (hpi). For early infection stages (≤24 hpi), leaf discs (0.5 cm × 0.5 cm) were excised within 1 mm of the inoculation points. For later stages (48 and 72 hpi), samples were collected at the junction between diseased and healthy tissues.

Sample processing was conducted following a modified procedure described by Li et al. [33]: (1) Fixation/decolorization: Leaf discs were immersed in fixative solution (ethanol: glacial acetic acid = 3:1, *v*/*v*) at 4 °C for 48 h, with replacement at 24 h, to remove chlorophyll. (2) Clearing/staining: Discs were immersed in lactophenol solution (20% lactic acid, 20% phenol, 40% glycerol, and 20% water) for 2 h, followed by staining with 0.05% trypan blue in lactophenol at 4 °C for 24 h (fungal structures stain blue). (3) Rinsing/preservation: Leaf discs were rinsed three times (5 min per rinse) with sterile distilled water and stored at 4 °C in preservation solution (acetic acid–glycerol–water = 1:1:1, *v*/*v*/*v*).

Processed leaf samples were mounted on microscope slides with 50% glycerol, covered with coverslips, and observed under a light microscope at 400× magnification. Key infection stages (conidial germination, hyphal penetration, and tissue colonization) were photographed to reconstruct the pathogen’s infection process.

### 2.8. Statistical Analysis

In this experiment, colony diameters were measured using the cross method, and mycelial growth rates were calculated as follows: Growth rate = [(Colony diameter − Disc diameter)/Incubation days] [11]. A one-way analysis of variance (ANOVA) was conducted using SPSS version 27.0. A *p*-value < 0.05 was considered statistically significant. Graphs were generated using Origin 2024.

## 3. Results

### 3.1. Symptoms of Leaf Spot Disease on Impatiens hawkeri

In the early infection stage, small, light yellow-brown spots appeared on *I. hawkeri* leaves. As the infection progressed, these spots gradually expanded into circular or elliptical lesions. The lesion margins exhibited indistinct concentric rings and a brown coloration, while the central regions turned light brown. In severe cases, lesions merged, leading to extensive chlorosis, necrosis, and ultimately the death of the entire plant.

### 3.2. Pathogenicity Assay of the Pathogenic Fungus

**Figure 1:**
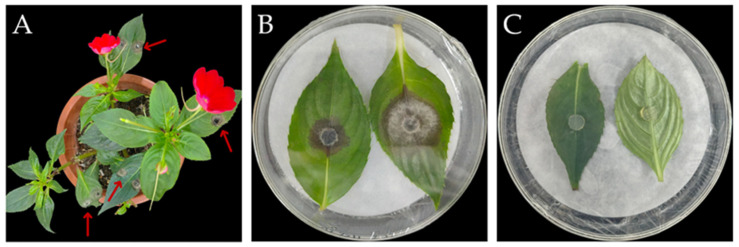
Symptoms of *E. multirostrata* development on the leaves of *I. hawkeri*: (**A**) symptoms on leaves of living plants after inoculation with strain IH-4; (**B**) symptoms on isolated leaves after inoculation with strain IH-4; (**C**) symptoms of isolated leaves after inoculation with sterile AGAR blocks.

Purified fungal strain IH-4 was subjected to pathogenicity testing via re-inoculation assays. At 2 days post-inoculation (dpi), leaves inoculated with IH-4 displayed distinct disease symptoms (Figure 1A,B), whereas control leaves inoculated with sterile PDA discs remained asymptomatic even at 7 dpi (Figure 1C). The fungal pathogen was successfully re-isolated from diseased leaves, and the re-isolated strain exhibited identical cultural and morphological characteristics as the original IH-4 strain. Therefore, based on Koch’s postulates, strain IH-4 was confirmed as the causal agent of leaf spot disease in *I. hawkeri*.

### 3.3. Morphological Observation of the Pathogenic Fungus

**Figure 2:**
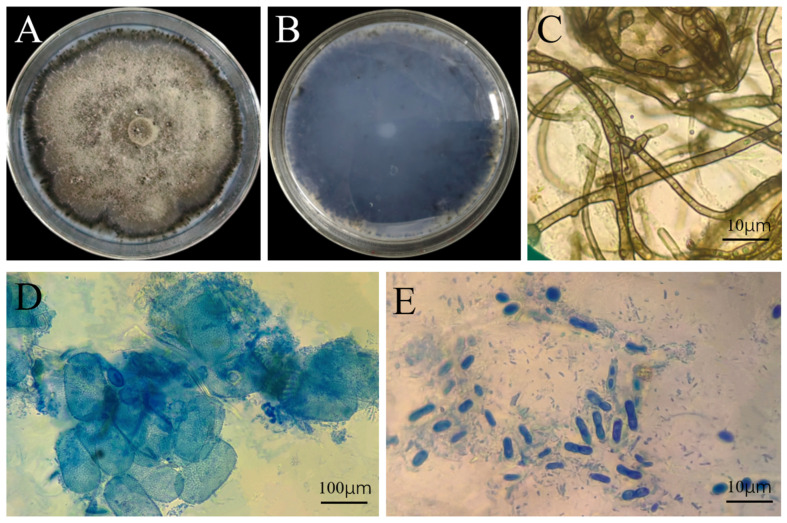
Morphological characteristics of IH-4 (*Ectophoma multirostrata)* on PDA: (**A**) colony front on PDA medium; (**B**) colony reverse on PDA medium; (**C**) hypha morphology; (**D**) conidiomata; (**E**) spore morphology.

After 7 days of incubation on PDA at 25 °C in the dark, colonies of *Ectophoma multirostrata* were circular, with floccose aerial hyphae that initially appeared white and later turned grayish-white to pale gray-green; colonies had a distinct dark brown to black marginal pigment zone, a yellowish-brown to dark brown reverse, and developed minute black pycnidia on the surface in older cultures. Strain IH-4 grew relatively slowly, requiring approximately 10 days to fully cover a 90 mm diameter Petri dish (Figure 2A,B).

The hyphae are septate, colorless to pale yellowish-brown, with uniform diameter, mostly undulate, and acutely branched. In some areas, hyphae intertwine and aggregate to form pseudosclerotium-like compact structures, and granular inclusions are visible within the cells (Figure 2C). Pycnidia were epigenous, solitary or gregarious, spherical to subglobose, 80–180 μm in diameter, with a dark brown wall composed of several layers of pseudoparenchymatous cells (Figure 2D). A papillate ostiole was visible on the mature pycnidia. Conidia were unicellular, hyaline, fusiform to ellipsoidal, and smooth-walled, measuring 4.2–7.2 μm × 1.2–2.5 μm (Figure 2E). These observations indicated that the cultural and morphological characteristics of strain IH-4 were consistent with those described for *E. multirostrata* [49].

### 3.4. Molecular Identification of the Pathogenic Fungus

**Figure 3:**
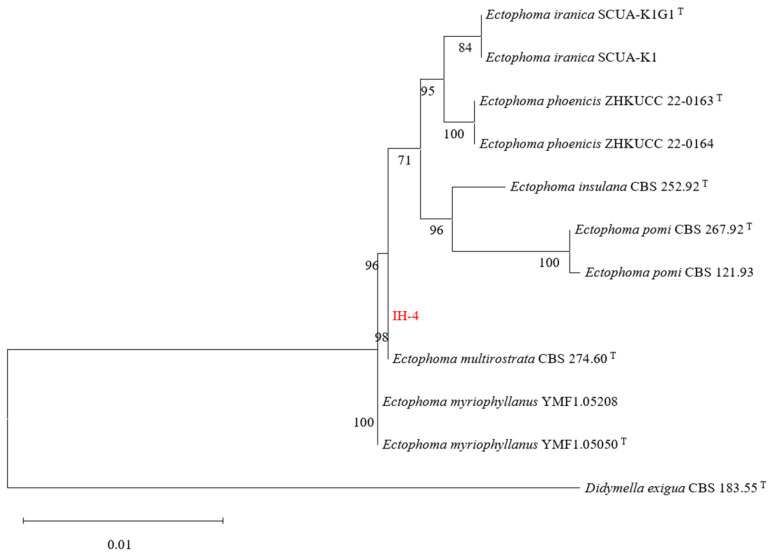
Phylogenetic tree constructed using the maximum likelihood method based on the concatenated ITS–LSU–*rpb2*–*tub2* sequences. The Tamura-Nei 93 (TN93) + Gamma distribution (+G) model was selected as the best-fitting nucleotide substitution model according to the Akaike Information Criterion (AIC). Branch support was assessed with 1000 bootstrap replicates; bootstrap values ≥ 50% are shown at the nodes. The scale bar represents the number of substitutions per site.

PCR amplification of the ITS, LSU, *rpb2*, and *tub2* gene regions from strain IH-4 yielded sequences that have been deposited in GenBank under accession numbers PX974131 (ITS), PX974132 (LSU), PX981988 (*rpb2*), and PX981989 (*tub2*), respectively. A BLASTn search based on sequence similarity revealed that strain IH-4 shared 99% similarity with *E. multirostrata*, consistent with the morphological identification. To further confirm its taxonomic status, a phylogenetic analysis was performed using a concatenated dataset of the four gene regions (ITS, LSU, *rpb2*, and *tub2*). Reference sequences from type strains were obtained from curated fungal databases. In the resulting phylogeny (Figure 3), strain IH-4 clustered robustly (bootstrap support: 98%) with *E. multirostrata*, definitively identifying IH-4 as the causal agent of leaf spot disease in *I. hawkeri*. The sequences of strains used for phylogenetic tree construction are provided in the Supplementary Materials (Table S2).

### 3.5. Biological Characteristics of Strain IH-4

#### 3.5.1. Effect of pH on Mycelial Growth

**Figure 4:**
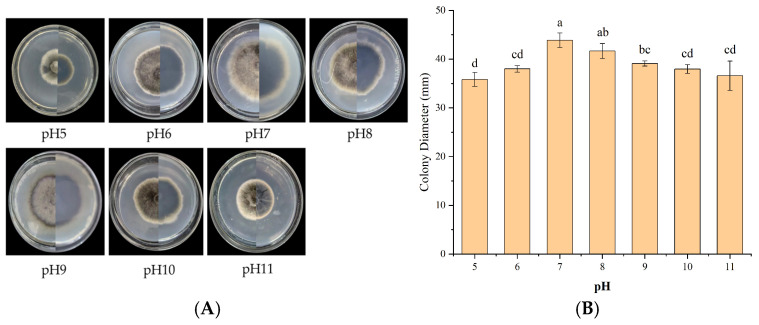
Growth of strain IH-4 under different pH values: (**A**) colony morphology: obverse side (**left**) and reverse side (**right**); (**B**) 5 d culture colony diameter. Different letters indicate significant differences at *p* < 0.05 according to ANOVA.

Strain IH-4 grew at pH 5–11, with optimal growth at pH 7 (colony diameter: 43.88 mm, mean ± SD, *n* = 3). Growth was inhibited at pH 5 (35.84 mm) and pH 11 (36.60 mm), indicating optimal growth under neutral conditions. The growth rate order was pH 7 > 8 > 9 > 6 > 10 > 11 > 5 (Figure 4). Growth at pH 7 significantly differed from growth at pH 5 and 11.

#### 3.5.2. Effect of Carbon Sources on Mycelial Growth

**Figure 5:**
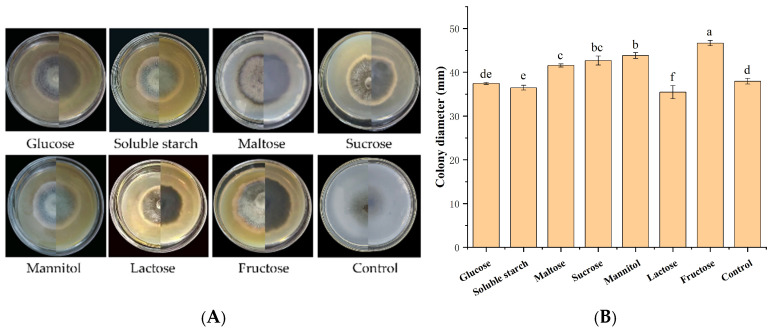
Growth of strain IH-4 under different carbon sources: (**A**) colony morphology: obverse side (**left**) and reverse side (**right**); (**B**) 5 d culture colony diameter. Different letters indicate significant differences at *p* < 0.05 according to ANOVA.

Fructose was the optimal carbon source (colony diameter: 46.68 mm), while lactose was the least favorable (35.48 mm) (Figure 5). Carbon sources significantly influenced growth. Growth rate ranking: fructose > mannitol > sucrose > maltose > control > glucose > soluble starch > lactose.

#### 3.5.3. Effect of Nitrogen Sources on Mycelial Growth

**Figure 6:**
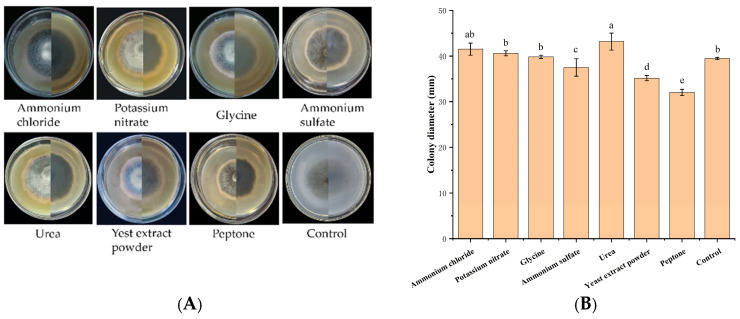
Growth of strain IH-4 under different nitrogen sources: (**A**) colony morphology: obverse side (**left**) and reverse side (**right**); (**B**) 5 d culture colony diameter. Different letters indicate significant differences at *p* < 0.05 according to ANOVA.

Urea was the optimal nitrogen source (colony diameter: 43.17 mm), while peptone resulted in the slowest growth (32.02 mm) (Figure 6). Nitrogen sources significantly affected mycelial growth. The growth rate ranking was urea > ammonium chloride > potassium nitrate > glycine > control > ammonium sulfate > yeast extract powder > peptone.

#### 3.5.4. Effect of Temperature on Mycelial Growth

**Figure 7:**
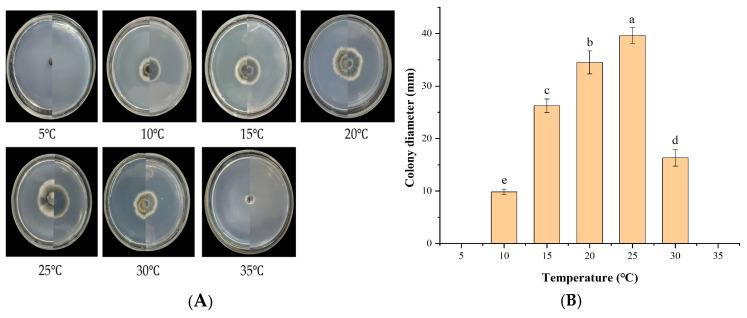
Growth of strain IH-4 under different temperatures: (**A**) colony morphology: obverse side (**left**) and reverse side (**right**); (**B**) 5 d culture colony diameter. Different letters indicate significant differences at *p* < 0.05 according to ANOVA.

IH-4 grew normally only between 10 and 30 °C (Figure 7). At 10 °C, growth was minimal (9.83 mm at 7 dpi). Growth increased with temperature, peaking at 25 °C (39.61 mm). Beyond 30 °C, growth was significantly reduced and ceased at higher temperatures. The optimal growth temperature was determined to be 25 °C, with limited tolerance for temperature fluctuations.

#### 3.5.5. Effect of Light Conditions on Mycelial Growth

**Figure 8:**
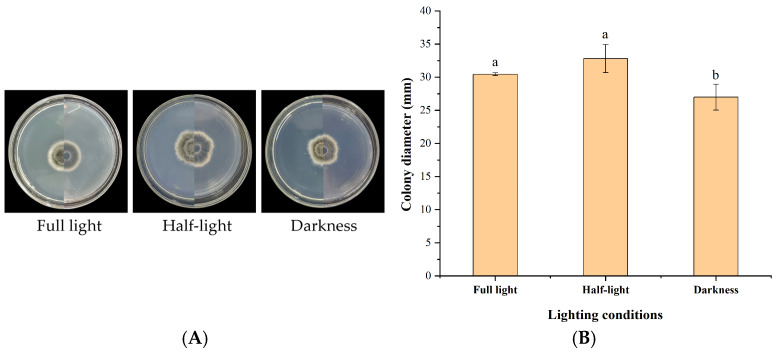
Growth of strain IH-4 under different light conditions: (**A**) colony morphology: obverse side (**left**) and reverse side (**right**); (**B**) 5 d culture colony diameter. Different letters indicate significant differences at *p* < 0.05 according to ANOVA.

IH-4 grew robustly under all tested light conditions (full light, semi-light [12 h light/12 h dark], and full darkness), with minimal differences observed (Figure 8). After 7 days, colony diameters were 30.44 mm (full light), 32.81 mm (semi-light), and 26.99 mm (darkness). Light conditions had no significant impact on mycelial growth.

#### 3.5.6. Effect of Culture Media on Mycelial Growth

**Figure 9:**
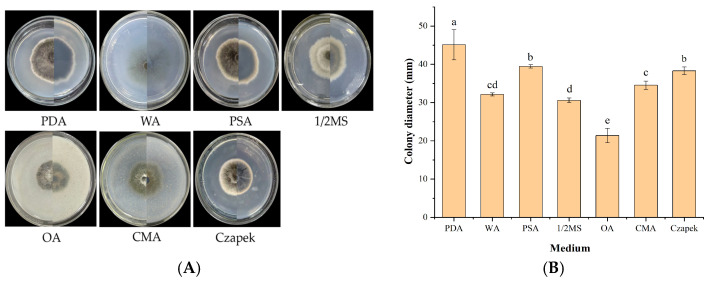
Growth of strain IH-4 under different media: (**A**) colony morphology: obverse side (**left**) and reverse side (**right**); (**B**) 5 d culture colony diameter. Different letters indicate significant differences at *p* < 0.05 according to ANOVA.

IH-4 growth varied among the 7 media tested (Figure 9). Mycelia grew most vigorously on PDA (producing long hyphae), while growth was slower on OA (short hyphae) and sparse on WA. Optimal media were PDA (45.12 mm) and PSA (39.45 mm at day 7). Unfavorable media included WA (32.13 mm), 1/2 MS (30.58 mm), and OA (21.37 mm at day 7). Nutrient composition significantly influenced growth, with PDA being the most suitable medium for strain IH-4.

### 3.6. Fungicide Sensitivity Assay of the Pathogenic Fungus

Eight chemical fungicides (Table 2) were evaluated in vitro by incorporating them into PDA medium at various concentrations. All fungicides tested exhibited varying degrees of inhibition against strain IH-4 (Table 4).

Among these, 25% azoxystrobin (SC) showed the greatest inhibitory effect (EC_50_: 0.0724 μg/mL), followed by 50% prochloraz–manganese complex (WP, EC_50_: 0.1778 μg/mL), 10% difenoconazole (EC, EC_50_: 0.1884 μg/mL), 10% polyoxins (WP, EC_50_: 3.3884 μg/mL), 25% carbendazim (WP, EC_50_: 3.8019 μg/mL), 40% myclobutanil (EC, EC_50_: 4.7863 μg/mL), 60% pyraclostrobin + metiram (WG, EC_50_: 20.8925 μg/mL), and 80% mancozeb (WP, EC_50_: 39.8107 μg/mL).

Probit regression analyses (SPSS 27.0) revealed fungicide regression slopes ranging from 0.47 to 1.7 (Table 4). 25% azoxystrobin had the steepest slope (1.7), indicating consistent sensitivity of *E. multirostrata* to this fungicide (higher slope = less variability in pathogen response).

Based on a comprehensive assessment of inhibition patterns and toxicological parameters (EC_50_, slope), 25% azoxystrobin demonstrated the strongest antifungal efficacy against strain IH-4.

### 3.7. Germination Process of Pathogenic Fungus Conidia

**Figure 10:**
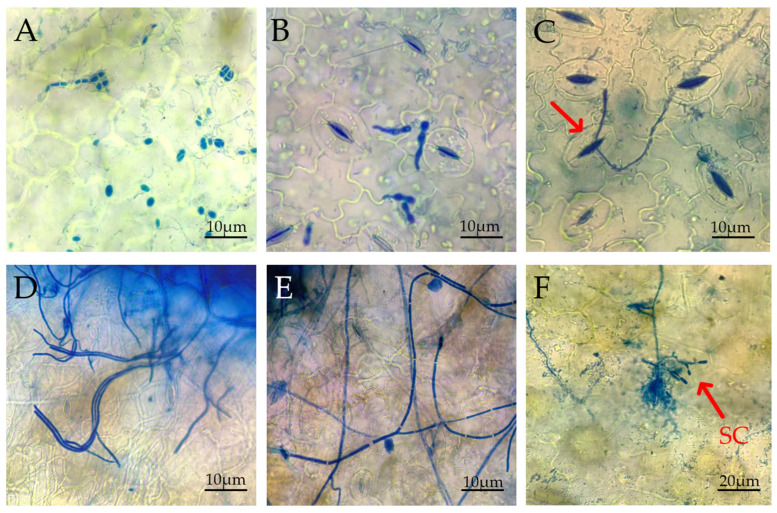
Microscopic observation of the infection process of strain IH-4. (**A**–**F**) The status of leaves at 0 h, 6 h, 12 h, 24 h, 48 h, and 72 h after inoculation, respectively. SC: secondary conidia.

Under optimal conditions (28 °C, 85% RH), consistent with infection assays, the germination dynamics of strain IH-4 conidia on *I. hawkeri* leaves were observed at multiple time points (Figure 10A–F; 400× magnification).

At 6 hpi, conidia initiated germination, producing germ tubes either bilaterally or laterally from the conidial ends (Figure 10A). By 12 hpi, germ tubes extended towards leaf stomata, with their tips aligning precisely with stomatal openings (Figure 10B). At 24 hpi, hyphae successfully penetrated through stomatal pores into leaf tissues, indicating completion of initial infection (Figure 10C).

At later stages (48–72 hpi), extensive hyphal branching occurred, forming a complex reticular network across the leaf surface. Secondary conidia emerged from hyphal tips, potentially facilitating further pathogen dispersal (Figure 10D–F).

## 4. Discussion

In this study, through isolation, morphological observation, molecular identification, and verification by Koch’s postulates, *E. multirostrata* was identified for the first time as the causal agent of leaf spot disease on *Impatiens hawkeri*. *Ectophoma multirostrata* is a globally distributed fungal species inhabiting diverse ecosystems and belongs to the phylum Ascomycota, class Dothideomycetes, subclass Pleosporomycetidae, order Pleosporales, and family Didymellaceae [50]. The Didymellaceae family was established by Gruyter in 2009, initially comprising three genera: *Ascochyta*, *Didymella*, and *Phoma* [51]. Notably, *E. multirostrata* was initially classified within the genus *Phoma* but was later reassigned to the new genus *Ectophoma* by Valenzuela-Lopez based on morphological and phylogenetic analyses [49]. In China, plant diseases reported to be caused by *E. multirostrata* include citrus greening disease on *Citrus reticulata* ‘Wogan’ [52], leaf spot in soybean seedlings exhibiting yellowing [53], rhizosphere fungal infections causing root rot in strawberries under continuous cropping [54], and bud blight in *Dendrobium officinale* [55]. However, reports on leaf spot diseases specifically induced by *E. multirostrata* remain limited, with no documented pathogenic role affecting *I. hawkeri*. This discovery not only expands the known host range of *E. multirostrata* but also provides essential etiological information for the precise control and management of *I. hawkeri* leaf spot disease. The results obtained here further validate the scientific basis of this taxonomic revision and add to the record of pathogenicity in ornamental plants.

Fungi within the family Didymellaceae are widely distributed across diverse habitats, including soil, air, oceans, and karst caves in Guizhou, China [56], as well as artificial substrates such as cement, asbestos, and pottery [57,58], and animal hosts, human tissues, and excreta [59,60,61]. According to the MycoBank database, the Didymellaceae family currently comprises over 5400 species across 39 genera [62], indicating its high species diversity. Within this family, *Didymella segeticola* exhibits optimal mycelial growth at temperatures ranging from 20 to 25 °C and pH values between 6 and 10. Its preferred carbon and nitrogen sources are lactose and peptone, respectively, and growth is promoted under alternating light and dark conditions with adequate ventilation [7]. *Alternaria tenuissima* is a widely distributed filamentous fungus that grows optimally at 25 °C and pH 5–6, with maximum sporulation achieved under alternating 12 h light and 12 h dark cycles [8]. For *Corynespora cassiicola*, the suitable temperature range for mycelial growth is 20–28 °C, while spore germination is favored at 24–32 °C. The optimal pH for growth lies between 6 and 10. Alternating light and dark conditions support both mycelial growth and sporulation, whereas continuous light inhibits mycelial development [63].

Research on the biological characteristics of *E. multirostrata* in China remains at an early stage. Existing evidence suggests that environmental factors—including medium composition (carbon and nitrogen sources), pH, temperature, and illumination—directly influence the pathogen’s survival and growth. Among these, a neutral pH environment coupled with optimal carbon and nitrogen sources significantly promotes pathogen proliferation, thereby influencing disease severity. Temperature, as a critical climatic factor, governs both disease onset timing and dissemination speed and indirectly regulates disease interannual occurrence patterns by affecting pathogen infectivity and overwintering survival. In contrast, illumination primarily exerts indirect effects by modifying host resistance, thereby influencing disease outcomes. The complex interplay of these environmental factors underlies the experimental conditions selected for this study, highlighting the necessity of integrating multiple dynamic factors for accurate field disease prediction. Nevertheless, further studies are required to explore the combined impact of multiple environmental factors in natural conditions and to examine the consequences of long-term environmental changes, such as temperature fluctuations associated with climate change, on pathogen behavior.

Previous studies have investigated the fungicide sensitivity of various pathogens associated with leaf spot diseases across different plant species, revealing that the most effective fungicides vary depending on the specific pathogen–host system. For instance, in controlling *Cercospora beticola*, the causal agent of sugar beet leaf spot, the combination of difenoconazole and tebuconazole exhibited the strongest efficacy [64]. For leaf spot of *Paris polyphylla* var. *chinensis*, 10% polyoxin wettable powder (EC_50_ = 11.921 mg/L) and 80% ethylicin emulsifiable concentrate (EC_50_ = 13.511 mg/L) demonstrated the highest antifungal activity [8]. In pecan (*Carya illinoinensis*), 450 g/L of prochloraz aqueous emulsion showed the strongest inhibition against *Fusarium commune*, *Botryosphaeria ribis*, and *Botrytis cinerea*, while 250 g/L of pyraclostrobin suspension concentrate was most effective against *Phoma fuckelii* [65]. Additionally, in studies on potato leaf spot in South Korea, 80% mancozeb and 25% difenoconazole were identified as the most effective fungicides for suppressing fungal growth [66].

Fungicide screening assays demonstrated that 25% azoxystrobin exhibited the strongest inhibitory effect on the mycelial growth of *E. multirostrata*, identifying it as a promising candidate for field control. However, the preliminary screening performed in this study included only 8 chemical fungicides, thus limiting its comprehensiveness. Given that the prolonged use of a single fungicide can easily lead to resistance development in agricultural practice, future research should broaden the scope of fungicide screening and establish multi-fungicide rotation programs coupled with field efficacy trials. In addition, environmental variability among regions (e.g., soil pH, temperature, humidity) and the simultaneous occurrence of diseases and pests may lead to regional variations in fungicide effectiveness. Therefore, further studies investigating the interactions among environmental conditions, fungicides, and pathogens are required to provide technical support for precise fungicide application. From a sustainable control perspective, future research should integrate biological control methods, agricultural practices, and chemical management to construct a multidimensional, integrated disease management system.

The directional elongation of germ tubes toward stomata following conidial germination, as observed in *E. multirostrata*, is a well-documented characteristic among stomata-penetrating pathogenic fungi. This precise stomatal targeting is mediated by thigmotropic and/or chemotactic responses to pathogens such as the rust fungus *Uromyces appendiculatus* and the downy mildew agent *Plasmopara viticola* [67]. Successful hyphal entry via stomata into leaf tissue indicates that *E. multirostrata* is capable of circumventing plant stomatal immunity. While it is established that bacteria such as *Xanthomonas campestris* utilize quorum sensing to secrete effectors that actively inhibit stomatal closure [68], analogous mechanisms may also be employed by this fungus. Furthermore, at 48–72 h post-inoculation (hpi), *E. multirostrata* developed an extensive hyphal network capable of generating secondary conidia. This capacity for secondary conidiation represents a key pathogenic trait, as it facilitates rapid inoculum amplification within favorable microenvironments and enhances field dispersal—a strategy also observed in pathogens such as *Colletotrichum musae* [69,70]. Collectively, these findings demonstrate that *E. multirostrata* combines efficient stomatal targeting and penetration with a rapid transition to secondary sporulation, providing a fundamental biological basis for the high pathogenicity and epidemic potential of the fungus.

This study is the first to confirm *E. multirostrata* as the pathogen causing leaf spot disease in *I. hawkeri*, enriching current knowledge regarding the pathogen’s host spectrum and associated symptomatology. The results from the environmental factor analyses and fungicide assays provide a theoretical basis for understanding disease occurrence patterns and developing emergency control measures. However, systematic research is still required to address scientific questions related to *E. multirostrata*, such as its overwintering mechanisms, transmission pathways, host-range expansion, and pathogenicity variations under environmental stresses. In particular, in the context of global climate change, fluctuations in critical factors such as temperature and humidity may alter pathogen adaptability, influencing disease epidemiology. Therefore, continuous monitoring of pathogen population dynamics and risk prediction using modeling approaches will be important areas for future research. Overall, this study provides technical support for safe *I. hawkeri* cultivation and offers a reference model for pathogen identification and disease management strategies in other ornamental plant diseases.

## 5. Conclusions

This study is the first to confirm *E. multirostrata* as the causal agent of leaf spot disease on *Impatiens hawkeri*, filling the gap in the etiology research of this disease. Through systematic analysis, the complete infection process of the pathogen—from conidial germination to the production of secondary conidia—was revealed, providing a theoretical basis for understanding the disease occurrence mechanism. Studies on biological characteristics identified the key environmental and nutritional factors affecting the pathogen’s growth, deepening the understanding of its ecological adaptability and offering references for predicting field disease epidemics and optimizing cultivation management. Fungicide sensitivity assessment screened out highly effective and stable control agents, providing technical support for constructing a precise disease control system and improving control sustainability. In summary, this study clarifies the core pathogenic characteristics and key control technologies of *I. hawkeri* leaf spot disease, offering important theoretical and practical support for scientific disease monitoring, early warning, and efficient control. It holds practical significance for safeguarding the development of related industries. Future research could focus on pathogenic genes, host resistance mechanisms, and fungicide synergism.

## Figures and Tables

**Table 2:** Characteristics of the 8 chemical fungicides tested.

Fungicides	Percent	Dosage Form	Manufacture
Difenoconazole	10%	Water-dispersible Granule (WDG)	Syngenta Nantong Crop Protection Co., Ltd., Nantong, China.
Polyoxins	10%	Wettable Powder (WP)	Sinochem Lihua (Tianjin) Agrochemicals Co., Ltd., Tianjin, China.
Carbendazim	25%	Wettable Powder (WP)	Sichuan Runer Technology Co., Ltd., Chengdu, China.
Azoxystrobin	25%	Suspension Concentrate (SC)	Syngenta Nantong Crop Protection Co., Ltd., Nantong, China.
Mancozeb	80%	Wettable Powder (WP)	Jinan Taihe Chemical Co., Ltd., Jinan, China.
Prochloraz–Manganese Complex	50%	Wettable Powder (WP)	FMC Suzhou Crop Protection Co., Ltd., Suzhou, China.
Pyraclostrobin + Metiram	60%	Water-dispersible Granule (WDG)	BASF (China) Co., Ltd., Shanghai, China.
Myclobutanil	40%	Wettable Powder (WP)	Shandong United Pesticide Industry Co., Ltd., Tai’an, China.

**Table 3:** Fungicide concentrations used for in vitro toxicity assays against fungal mycelial growth.

Fungicides	Concentrations of Active Ingredients (μg/mL)				
10% Difenoconazole [45]	0.2	0.4	0.5	1.0	1.5
10% Polyoxins [46]	0.5	1	2	4	8
25% Carbendazim [46]	1	2	4	8	16
25% Azoxystrobin [45]	0.03	0.04	0.05	0.06	0.07
80% Mancozeb [46]	2	4	8	12	36
50% Prochloraz–Manganese Complex [47]	0.01	0.03	0.09	0.27	0.81
60% Pyraclostrobin + Metiram [45]	0.25	0.5	1	2	4
40% Myclobutanil [48]	0.02	0.2	0.4	2	4

**Table 4:** Virulence regression equations and EC_50_ values of 8 fungicides against the mycelial growth of the pathogen.

Fungicides	Regression Equation	R^2^	EC_50_ (μg/mL)	95% CL
10% Difenoconazole	Y = 0.8X + 5.58	0.974	0.1884	0.72–0.877
10% Polyoxins	Y = 1.2X + 4.36	0.924	3.3884	0.996–1.408
25% Carbendazim	Y = 1.56X + 4.1	0.956	3.8019	1.356–1.758
25% Azoxystrobin	Y = 1.7X + 6.94	0.946	0.0724	1.458–1.944
80% Mancozeb	Y = 1.16X + 3.14	0.904	39.8107	0.933–1.385
50% Prochloraz–Manganese Complex	Y = 0.69X + 5.52	0.976	0.1778	0.623–0.752
60% Pyraclostrobin + Metiram	Y = 0.47X + 4.38	0.900	20.8925	0.374–0.569
40% Myclobutanil	Y = 0.57X + 4.61	0.936	4.7863	0.479–0.657

## Data Availability

The original data presented in this study are included in the article. The ITS, LSU, *rpb2*, and *tub2* gene sequences generated in this study via Sanger sequencing have been deposited in the GenBank database of the National Center for Biotechnology Information (NCBI) under the accession numbers PX974131, PX974132, PX981988, and PX981989, respectively. All other reagents, microbial materials, and datasets used, created, and analyzed in the study are available upon reasonable request to the corresponding author.

## Supplementary Materials

The following supporting information can be downloaded at: https://www.mdpi.com/article/10.3390/jof12030210/s1. Table S1: Formulations of culture media; Table S2: Reference strains used for phylogenetic analysis.
