# Supplementary material for: Studies on Pathogen Identification, Biological Characteristics and Fungicide Sensitivity of Impatiens hawkeri Leaf Spot Disease
Source: J Fungi (Basel). 2026 Mar 14;12(3):210. doi: 10.3390/jof12030210 (PMC13027522; doi:10.3390/jof12030210)
Supplement: Supplementary file 1 [file jof-12-00210-s001.zip › jof-4172067-supplementary.pdf]

Table S1. Formulations of culture media.

Medium Type	Formulation
PDB	1 L Sterile water, 200 g potato, 20 g glucose
PDA	1 L Sterile water, 200 g potato, 20 g glucose, 20 g agar
PSA	1 L Sterile water, 200 g potato, 20 g sucrose, 20 g agar
WA	1 L Sterile water, 20 g agar
1/2MS	1 L Sterile water, 1.65 g NH_4NO_3； 1.90 g KNO_3； 0.44 g $CaCl_2·2H_2O$ ； 0.37 g $MgSO_4·7H_2O$； 0.17 g KH_2PO_4； 6.2 mg H_3BO_3； 22.3 mg $MnSO_4·4H_2O$； 8.6 mg $ZnSO_4·7H_2O$； 0.83 mg KI； 0.25 mg $Na_2MoO_4·2H_2O$； 0.025 mg $CuSO_4·5H_2O$； 0.025 mg $CoCl_2·6H_2O$； 27.8 mg $FeSO_4·7H_2O$； 37.3 mg $Na_2EDTA·2H_2O$； 100 mg myo-inositol； 0.5 mg nicotinic acid； 0.5 mg pyridoxine HCl； 0.1 mg thiamine HCl； 2 mg glycine； 30.0 g sucrose
OA	1 L Sterile water, 30 goat, 20 g agar
CMA	1 L Sterile water, 5 g cornmeal, 20 g agar
CDA	1 L Sterile water, 200 g potato,3.0 g $NaNO_3$, 0.5 g KCl, 30.0 g sucrose, 0.5 g $MgSO_4·7H_2O$, 0.01 g $FeSO_4·7H_2O$, 1.0 g K_2HPO_4, 15.0 g agar

Table S2. Reference strains used for phylogenetic analysis.

Latin name	Strain number	Accession number			
		**ITS**	**LSU**	***rpb2***	***tub2***
E. multirostrata	IH-4	PX974131	PX974132	PX981988	PX981989
E. multirostrata	CBS 274.60	FJ427031	GU238111	LT623265	FJ427141
E. insulana	CBS 252.92	MN973481	MN943685	MT018070	MT005581
E. iranica	SCUA-K1G1	MK519382	MK519389	-	MK519562
E. iranica	SCUA-K1	MK519381	MK519388	-	MK519561
E. phoenicis	ZHKUCC 22-0163	OQ275208	OQ275194	OQ343375	OQ336259
E. phoenicis	ZHKUCC 22-0164	OQ275209	OQ275195	OQ343376	OQ336260
E. pomi	CBS 267.92	-	GU238128	LT623263	GU237643
E. pomi	CBS 121.93	MN972933	MN973320	MN983570	MN983948
E. myriophyllanus	YMF 1.05208	MH257418	MH257503	MH311849	MH423014
E. myriophyllanus	YMF 1.05050	MH257417	MH257502	MH311848	MH423013
Didymella exigua	CBS 183.55	GU237794	EU754155	EU874850	GU237525
